# The short-term effects of selective dorsal rhizotomy on gait compared to matched cerebral palsy control groups

**DOI:** 10.1371/journal.pone.0220119

**Published:** 2019-07-30

**Authors:** Rory O’Sullivan, Jane Leonard, Aoife Quinn, Damien Kiernan

**Affiliations:** Central Remedial Clinic, Clontarf, Dublin, Ireland; Boston Children's Hospital / Harvard Medical School, UNITED STATES

## Abstract

**Objectives:**

To examine the short-term effects of selective dorsal rhizotomy (SDR) ± soft-tissue surgery on gait in cerebral palsy (CP) compared to matched controls with no surgical intervention.

**Methods:**

Participants had gait analysis before and one year after SDR. Non SDR participants were retrospectively matched for age and all significant gait parameters. The SDR group was further subdivided into those who had concomitant orthopaedic surgery and those who had SDR only.

**Results:**

The SDR group consisted of 29 participants (mean age 5.8 years at baseline, 7.7 years at follow-up). Of these, 13 had concomitant orthopaedic surgery. The non SDR group consisted of 18 participants (mean age at baseline 6.1 years, 8.1 years at follow-up). SDR ± soft-tissue surgery significantly improved step-lengths, knee flexion at initial contact and mid-stance, ankle dorsiflexion, foot progression and timing of peak knee flexion. None of these improvements in gait were seen without surgical intervention. While more improvements were seen in those who had SDR and orthopaedic surgery, SDR only resulted in improved step-lengths, knee extension, foot progression and timing of peak knee flexion.

**Conclusions:**

SDR ± soft-tissue surgery results in short-term improvements in gait which are not seen without surgical intervention. While those who had SDR and soft-tissue surgery demonstrated more changes in gait, many improvements were attributable to SDR only.

## Introduction

Selective dorsal rhizotomy (SDR) is a permanent surgical procedure which has been shown to effectively reduce spasticity associated with cerebral palsy (CP) by selective sectioning of the lumbosacral afferent nerve rootlets [1–3]. However, there is limited evidence that this reduction in spasticity significantly improves activity and function compared to other treatment options. Tedroff found that while the spasticity-reducing effects of SDR were pronounced, this did not improve long-term functioning or prevent contractures which highlighted that contracture development in CP is not mediated by spasticity alone [4]. A meta-analysis of three randomized clinical trials confirmed a change in spasticity, with only a small, yet statistically significant advantage of SDR and physiotherapy compared to physiotherapy only with respect to the impact on gross motor function [3]. A systematic review summarised that there is poor to moderate evidence that SDR has a positive long-term effect on the international classification of functioning, disability and health (ICF) body structure and function domains with no evidence that SDR has a positive long-term influence on the ICF activity or participation domains [5].

The goal of SDR in those with ambulant CP is often to improve gait. However, the evidence for improvement in gait post SDR is variable. A systematic review has suggested that SDR is effective in improving gait kinematics but noted the quality of evidence was low[2]. Only a limited number of studies have examined changes in gait kinematics in detail following SDR [6–8]. While these studies report improvements in gait kinematics following SDR, the majority did not include a matched control group [6, 7]. As a result it is difficult to isolate the effects of SDR from natural development or alternative treatment approaches. Only one study compared outcomes following SDR to an appropriately matched cohort of CP participants undergoing ‘standard’ treatment [8]. These long-term follow-up results found that gait improved in both matched SDR and non-SDR groups and while the non-SDR group had a larger improvement in gait pathology, this was at the expense of significantly more orthopaedic interventions. However, by the time of long-term follow-up the SDR group had also had a mean of 10.8 orthopaedic interventions meaning that long-term outcomes in this group were due to both the initial SDR and subsequent orthopaedic intervention and the relative contribution of each could not be isolated.

SDR is currently not available in Ireland. However, recently children who meet recognised selection criteria [1, 9, 10] may be reviewed for suitability for this intervention. If felt appropriate they are referred to be assessed for SDR in a neighbouring country and a limited number then elect to proceed with surgery. This assessment is funded by the state, along with the SDR surgery. In addition, outside of the formal referral pathway, a small number of children and their families also self-fund travel to under-go SDR privately. As the national gait laboratory, we carry-out pre and post SDR assessments on all those assessed and referred through the funded pathway and on a large number of those choosing to pursue SDR independent of the national referral pathway. The absence of an SDR service in this country means that the majority of children with CP would not presently be offered this, including those who may otherwise have met common selection criteria. This allows us to compare SDR outcomes to matched CP controls who did not have SDR.

The primary aim of this study is to examine whether SDR leads to short-term improvements in gait kinematics compared to a matched control group who had no surgical intervention. In addition, the outcomes of SDR only compared to SDR with orthopaedic surgery at or about the same time will be examined separately. Our hypothesis was that those who had SDR would demonstrate improvements in gait beyond those seen in those who had no surgery.

## Method

### Compliance with ethical standards

In this retrospective study, medical records of human patients were reviewed after approval was obtained from the institutional review board. Parents/guardians of all patients provided written consent for data to be used for research purposes. All data were anonymised on extraction prior to any analysis and use in this study.

### Participants

Appropriate local institutional approval was obtained for this retrospective cohort study.

Participants were included in the SDR group if they met the following criteria: (1) diagnosis of bilateral, spastic CP, GMFCS II-III; (2) pre and post-operative three-dimensional gait analysis completed at our gait laboratory; (3) underwent SDR between 2005 and 2017; (4) age 4 to 14 years at time of SDR.

The non-SDR group were identified retrospectively by searching our gait laboratory database from 1998 onwards. Participants were first matched for age, diagnosis, and BMI. Participants were then identified who matched the SDR group at baseline for normalised gait speed (normalised to height), normalised step length (normalised to height) and all key gait kinematic variables (Table 1) that were seen to significantly change in the SDR group.

**Table 1:** Summary of clinical examination and gait measures in the SDR and No Surgery groups at baseline and follow-up analyses.

	SDR (n = 29)			No Surgery (n = 18)		
	Baseline	Follow-up	p-value	Baseline	Follow-up	p-value
Males/Females	17/12	17/12		12/6	12/6	
Age (years) (Mean(SD))	5.8 (2.2)	7.7 (2.5)		6.1 (1.2)	8.1 (1.5)	
Age at Surgery (years) (Mean (SD))	6.4 (2.2)			N/A		
**Clinical Examination**						
Body Mass Index	15.9 (15.2–17.3)	16.0(15.2–17.8)	0.85	16.0(15.8–17.0)	16.3(15.5–17.1)	0.56
Hamstring Length(^0^)	60.0(50.0–65.0)	53.5(45.0–64.5)	<0.01^b^	55.0(50.0–64.3)	61.0(50.0–68.5)	0.02^**b**^
Gastrocnemius Length(^0^)	95.5(90.0–101.5)	105.0(95.0–101.0)	<0.01^b^	95.0(90.0–100.0)	95.0(90.0–100.0)	0.20
Rectus Femoris Length(^0^)	135.0(130.0–140.0)	135.0(120.0–140.0)	<0.01^b^	135.0(120.0–140.0)	130.0(115.0–140.0)	0.38
**Gait Data**						
Gait Deviation Index	62.7(55.1–68.8)	69.2(62.4–76.4)	<0.01^b^	68.5(60.0–72.7)^**a**^	70.3(61.0–76.9)	0.14
Normalised Gait Speed†	0.3(0.2–0.6)	0.5(0.2–0.7)	0.07	0.4(0.2-.06)	0.4(0.2–0.5)	0.42
Normalised Step Length†	0.2(0.2–0.3)	0.3(0.2–0.3)	<0.01^b^	0.3(0.2–0.3)	0.2(0.2–0.3)	0.55
Mean Pelvic Tilt(^0^)	16.5(11.8–22.2)	23.3(15.6–25.5)	0.02^b^	18.5(15.2–20.5)	17.4(15.3–19.4)	0.32
Hip Flexion/Extension Range(^0^)	42.0(35.4–48.9)	46.4(39.6–52.6)	0.04^b^	42.3(38.2–48.0)	39.5(32.8–44.1)	0.02^**b**^
Hip Flexion in Stance(^0^)	11.5(5.3–16.8)	7.6(2.1–15.5)	0.16	10.1(7.4–16.0)	12.1(5.2–18.0)	0.85
Knee Flexion/Extension Range(^0^)	43.5(37.6–52.3)	53.1(45.3–59.7)	<0.01^b^	50.0(41.9–56.3)	44.5(30.7–49.1)	<0.01^**b**^
Peak Knee Flexion(^0^)	66.5(60.9–74.2)	63.8(58.5–72.9)	0.30	69.2(64.4–72.4)	59.8(54.3–64.9)	<0.01^**b**^
Time to Peak Knee Flexion (%GC)	85.0(81.3–89.8)	80.0(72.3–87.5)	<0.01^b^	85.0(82.0–88.0)	82.0(77.8–85.0)	0.05
Knee Flexion in Stance(^0^)	20.9(14.9–32.7)	10.3(2.1–18.9)	<0.01^b^	20.3(14.0–25.6)	20.9(10.8–27.7)	0.81
Knee Flexion at Ground Contact(^0^)	43.9(35.0–56.0)	27.1(19.3–35.3)	<0.01^b^	38.7(36.1–43.6)	38.5(33.5–43.4)	0.13
Ankle Plantar/Dorsi-Flexion Range(^0^)	27.0(19.8–35.3)	29.2(21.1–36.1)	0.94	31.3(22.6–36.8)	21.2(17.2–30.1)	<0.01^**b**^
Maximal Ankle Dorsiflexion(^0^)	10.8(-0.30–16.5)	13.7(5.9–17.1)	<0.01^b^	11.5(2.5–17.6)	10.3(0.1–18.7)	0.58
Foot Progression Angle‡(^0^)	-4.0(-19.1–9.7)	-14.5(-25.8- -5.7)	<0.01^b^	-4.7(-17.9–4.8)	-6.1(-15.8–0.6)	0.58
Mean Hip Rotation‡(^0^)	2.2(-3.7–8.3)	3.3(-4.2–10.1)	0.43	6.4(0.6–12.8)^**a**^	6.6(-2.6–11.3)	0.71
Mean Tibia Rotation‡(^0^)	-5.0(-9.5–1.0)	-18.3(-24.5- -5.7)	<0.01^b^	-4.6(-10.7–3.9)	-10.0(-18.7- -1.8)	0.05

Data are Median (Interquartile Range).

^**a**^ p<0.05 for differences between groups at baseline (Mann–Whitney U test).

^**b**^ p<0.05 for baseline to follow-up changes within group (Wilcoxon signed rank test).

SDR—selective dorsal rhizotomy

N/A—Not Applicable

GDI—Gait Deviation Index; GC—Gait Cycle.

†Normalised to height.

‡ Rotation values: positive values internal; negative values external.

All study participants had a baseline and follow-up gait analysis. For the SDR group the baseline analysis was the last assessment before SDR and the follow-up was the post-operative analysis. For the non-SDR group the baseline and follow-up analyses were those that best matched the age and follow-up time between pre and post SDR analyses. The non-SDR group had no surgical intervention (SDR or orthopaedic) between baseline and follow-up analyses.

### Treatment

In addition to SDR, details on orthopaedic surgical intervention occurring between baseline and follow-up analysis were obtained from medical files. As all surgical treatments in the SDR group were carried out abroad the exact surgical technique was often not available. Therefore soft-tissue orthopaedic surgery is reported at the level of the muscle group (e.g. hamstring/calf-complex/psoas etc.). Each individual orthopaedic intervention was counted as one instance e.g. bilateral hamstrings were counted as two instances [8].

### Gait analysis and clinical examination

All participants had a barefoot three-dimensional gait analysis and related clinical examination in our laboratory. All three-dimensional kinematic data were captured using a Codamotion active marker system (Charnwood Dynamics, Leicestershire, UK) using a modified Helen Hayes protocol at a capture rate of 200Hz [11]. Due to the central nature of SDR both limbs were included as per Van Campenhout et al [12]. As the pelvis is a single unit, kinematics of one side only were included in the analysis.

Clinical examination was completed by an experienced physiotherapist. Popliteal angle measure of hamstring length was recorded in supine with the contralateral hip and knee flexed to align the pelvis to neutral, the recorded measure was degrees from full extension. Gastrocnemius length was assessed with the knee extended and rectus femoris length was assessed as the maximum knee flexion in prone.

### Data analysis

Statistical analyses were conducted using Stata v13.1 software (StataCorp LP, USA) and with Statistical non-Parametric Mapping (SnPM) (SPM1d version 0.4, available for download at http://www.spm1d.org/) in MATLAB (The Mathworks Inc., Natick, M.A., 2015). All gait analysis and clinical measures are reported as median (interquartile range). Differences within groups were compared using a Wilcoxon signed-rank test while differences between groups at baseline (SDR–No Surgery; SDR only–SDR and Orthopaedic Surgery) were compared using a Mann-Whitney U-test. For the SnPM analyses, normality of data were assessed with a build in function in SPM (spm1d.stats.normality.ttest_paired). As all data did not follow a normal distribution it was decided to use SnPM on all kinematic variables. A non-parametric two-tailed two-sample t-test (SnPM {t}) was used to test differences between groups pre-operatively and post-operatively across the complete gait cycle. A significance level of p = 0.05 was used for all tests.

## Results

### Study cohort

The SDR group consisted of 29 participants (17 male; 12 female) with a mean age of 5.8 (2.2) years at baseline analysis, mean age of 7.7 (2.5) years at follow-up and mean age of 6.4 (2.2) years at SDR. Of the 29 SDR participants 16 had SDR only and 13 had orthopaedic surgery in addition to SDR. Of this 13, all orthopaedic interventions were soft-tissue releases and there were a total of 49 procedures (mean 3.77) consisting of 25 hamstring and 24 calf-complex. The non-SDR control cohort consisted of 18 participants (12 male; 6 female) with a mean age at baseline of 6.1 (1.2) years and mean age of 8.1 (1.5) years at follow-up.

### Clinical examination and gait parameters

**Fig 1:**
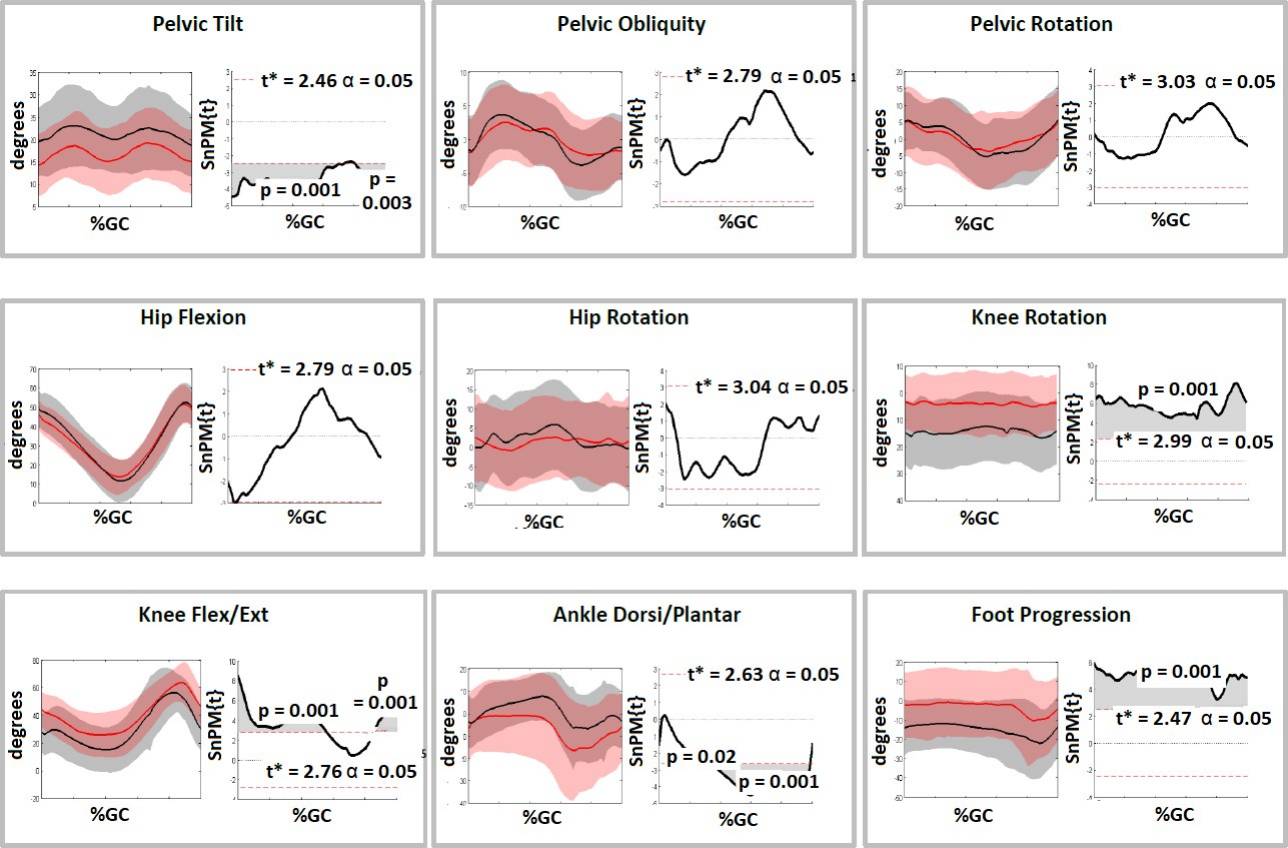
Comparison of gait kinematics pre and post operatively for SDR group (n = 29) and subsequent SnPM tests for the kinematic curves. Kinematic graphs show Pre SDR (Red), Post SDR (Black). Shaded grey areas on SnPM curves to the right highlight the period of the gait cycle where the kinematic curves differed significantly.

The SDR group demonstrated a number of significant changes in gait data at follow-up compared to baseline (Fig 1). Of note, Gait Deviation Index (GDI) (62.7 to 69.2, p<0.01), normalised step length (0.2 to 0.3, p<0.01), knee flexion/extension range (43.5^o^ to 53.1^o^, p <0.01), knee extension at initial contact (IC) (43.9^o^ to 27.1^o^, p<0.01), knee flexion in stance (20.9^o^ to 10.3^o^, p < 0.01), mean tibia rotation (-5.0^o^ to -18.3^o^, p<0.01) and foot progression angle (4.0^o^ to -14.5^o^, p < 0.01) all improved, while mean anterior pelvic tilt increased (16.5^o^ to 23.3^o^, p = 0.02) (Table 1). SnPM analysis was largely consistent with the findings above and confirmed increased anterior pelvic tilt, improved knee rotation and improved foot progression angle through-out the gait cycle. However, knee extension significantly improved only at initial contact and initial stance phase and ankle dorsiflexion only improved during late stance and swing phase (Fig 1). In addition, clinical examination measures of rectus femoris, gastrocnemius and hamstring lengths all differed at follow-up (Table 1).

The No Surgery group demonstrated a decreased dynamic hip and knee flexion/extension range during gait (42.3^o^ to 39.5^o^, p = 0.02 and 50.0^o^ to 44.5^o^, p<0.01 respectively). In addition, peak knee flexion and ankle plantar/dorsiflexion ranges were reduced at follow-up (Table 1). Hamstring length was the only clinical measure different at follow-up (55.0^o^ to 61.0^o^, p = 0.02).

**Fig 2:**
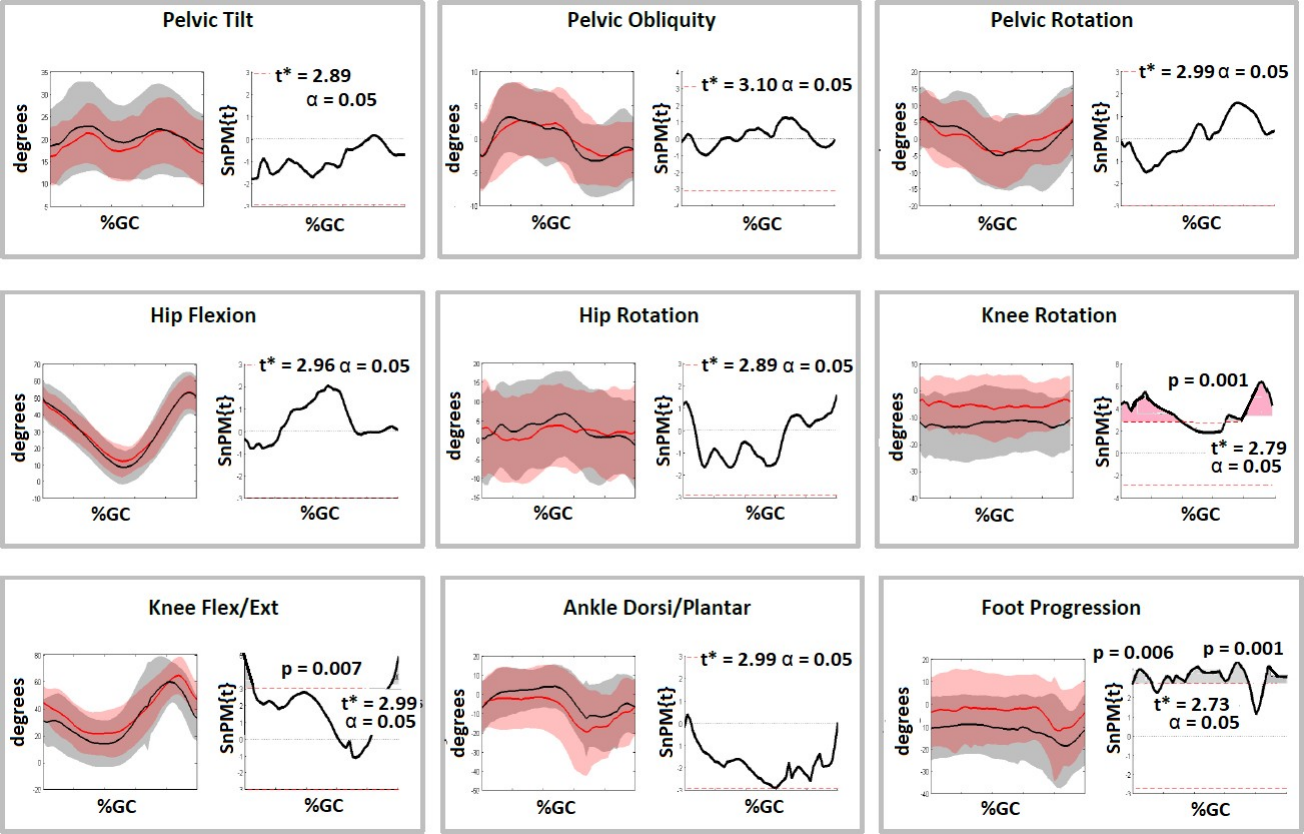
Comparison of gait kinematics pre and post operatively for SDR Only group (n = 16) and subsequent SnPM tests for the kinematic curves. Kinematic graphs show Pre SDR Only (Red), Post SDR Only (Black). Shaded grey areas on SnPM curves to the right highlight the period of the gait cycle where the kinematic curves differed significantly.

**Fig 3:**
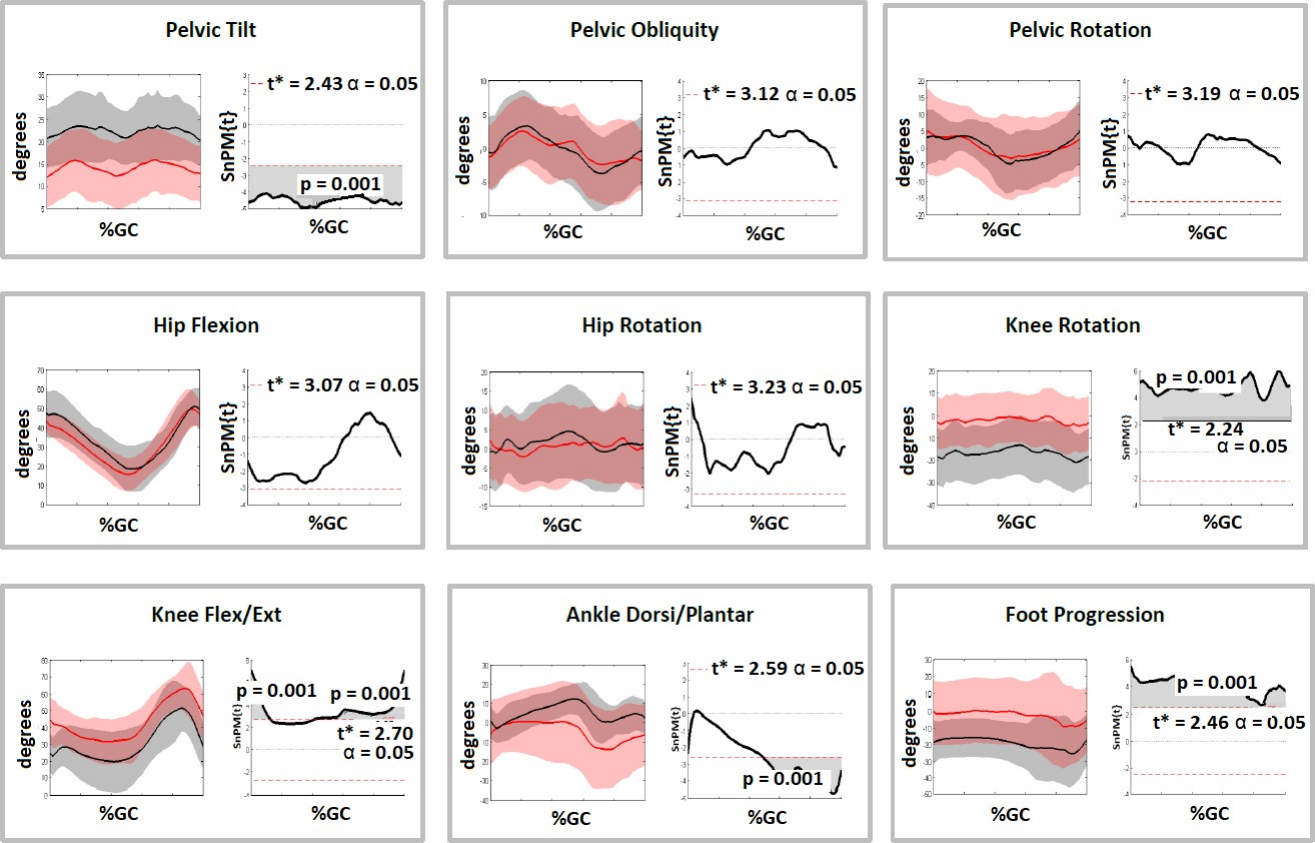
Comparison of gait kinematics pre and post operatively for SDR & Orthopaedic Surgery group (n = 13) and subsequent SnPM tests for the kinematic curves. Kinematic graphs show Pre SDR & Orthopaedic Surgery (Red), Post SDR & Orthopaedic Surgery (Black). Shaded grey areas on SnPM curves to the right highlight the period of the gait cycle where the kinematic curves differed significantly.

The SDR group was sub-divided into those who had concomitant orthopaedic surgery and those who had SDR only (Figs 2 & 3). A number of significant differences were present at follow-up for both groups (Table 2). Of note, the SDR-only group demonstrated improved normalised step length (0.2 to 0.3, p<0.01), improved knee extension at IC and stance (43.5^o^ to 33.1^o^, p<0.01 and 16.5^o^ to 11.6^o^, p = 0.03 respectively) and an improved foot progression angle (-6.5^o^ to -12.6^o^, p = 0.02). The SDR and Orthopaedic Surgery group demonstrated similar changes in addition to an increased dorsiflexion (13.0^o^ to 15.1^o^, p = 0.02) and reduction in peak knee flexion (65.1^o^ to 62.2^o^, p = 0.04). SnPM analyses of these groups highlighted that knee extension and ankle dorsi-flexion improved through more of stance phase when SDR was combined with orthopaedic surgery (Figs 2 and 3) though Table 2 highlights that this group were more flexed during gait at baseline. The full listing of results can be found in Table 2.

**Table 2:** Summary of clinical examination and gait measures in the SDR only and SDR and Orthopaedic Surgery groups at baseline and follow-up analyses.

	SDR Only (n = 16)			SDR and Orthopaedic Surgery (n = 13)		
	Baseline	Follow-up	p-value	Baseline	Follow-up	p-value
Males/Females	10/6	10/6		7/6	7/6	
Age (years) (Mean (SD))	5.5 (1.6)	7.3 (2.1)		6.2 (2.8)	8.3 (2.9)	
Age at Surgery (years) (Mean (SD))	6.1 (1.9)			6.7 (2.6)		
**Clinical Examination**						
Body Mass Index	15.9 (15.3–17.4)	15.6(15.1–16.9)	0.54	16.3(14.9–17.3)	16.9(15.2–18.3)	0.38
Hamstring Length(^0^)	60.0(53.8–63.5)	55.0(47.3–65.0)	0.02^**b**^	60.0(50.0–69.5)	50.0(45.0–60.0)	<0.01^**b**^
Gastrocnemius Length(^0^)	100.0(90.0–105.0)	102.5(92.8–110.0)	0.32	93.5(85.3–100.0)^**a**^	106.0(100.0–110.0)	<0.01^**b**^
Rectus Femoris Length(^0^)	135.0(130.0–140.0)	130.0(128.8–130.0)	<0.01^**b**^	135.0(130.0–140.0)	127.5(120.0–140.0)	0.04^**b**^
**Gait Data**						
Gait Deviation Index	62.5(56.1–68.4)	69.5(59.0–75.6)	0.08	62.8(52.7–71.2)	67.6(62.9–74.9)	0.07
Normalised Gait Speed†	0.3(0.2–0.6)	0.5(0.2–0.7)	0.08	0.3(0.1–0.5)	0.5(0.1–0.6)	0.15
Normalised Step Length†	0.2(0.2–0.3)	0.3(0.3–0.3)	<0.01^**b**^	0.2(0.1–0.3)	0.3(0.2–0.3)	<0.01^**b**^
Mean Pelvic Tilt(^0^)	17.5(13.1–22.4)	20.5(14.5–24.6)	0.49	15.1(9.0–17.9)	24.2(21.5–27.6)	<0.01^**b**^
Hip Flexion/Extension Range(^0^)	46.1(41.4–51.8)	47.1(42.7–55.1)	0.25	36.9(31.9–41.2)^**a**^	46.1(35.0–50.2)	0.02^**b**^
Hip Flexion in Stance(^0^)	10.5(4.3–14.1)	4.7(0.9–9.0)	0.06	15.4(9.0–20.0)^**a**^	12.0(6.5–19.2)	0.79
Knee Flexion/Extension Range(^0^)	49.0(40.8–55.9)	52.9(45.8–67.3)	0.02^**b**^	41.2(33.6–43.9)^**a**^	53.9(44.6–58.2)	<0.01^**b**^
Peak Knee Flexion(^0^)	70.5(60.3–77.5)	68.1(60.1–76.4)	0.56	65.1(61.2–73.1)	62.2(56.7–66.8)	0.04^**b**^
Time to Peak Knee Flexion (%GC)	85.0(82.8–89.0)	79.5(72.0–84.3)	<0.01^**b**^	83.5(76.8–89.8)	80.5(75.3–88.0)	0.42
Knee Flexion in Stance(^0^)	16.5(6.6–26.7)	11.6(0.1–21.9)	0.03^**b**^	25.1(19.5–40.4)^**a**^	9.6(3.5–16.8)	<0.01^**b**^
Knee Flexion at Ground Contact(^0^)	43.5(34.9–54.4)	33.1(20.3–39.6)	<0.01^**b**^	44.5(35.4–57.7)	22.7(18.2–28.8)	<0.01^**b**^
Ankle Plantar/Dorsi-Flexion Range(^0^)	31.3(24.1–41.1)	29.2(21.2–34.5)	0.08	24.0(17.5–31.3)^**a**^	29.0(20.8–37.3)	0.02^**b**^
Maximal Ankle Dorsiflexion(^0^)	5.7(-1.3–12.9)	9.6 (3.0–16.2)	0.13	13.0(3.1–17.5)	15.1(12.7–19.5)	0.02^**b**^
Foot Progression Angle‡(^0^)	-6.5(-13.2–6.6)	-12.6(-21.6- -3.5)	0.02^**b**^	2.7(-21.6–12.1)	-19.7(-30.3- -8.8)	<0.01^**b**^
Mean Hip Rotation‡(^0^)	2.9(-4.0–9.2)	3.7(-4.2–10.5)	0.61	0.9(-3.6–6.1)	3.1(-5.7–9.9)	0.69
Mean Tibia Rotation‡(^0^)	-4.5(-9.3–0.4)	-14.2(-22.6- -4.7)	<0.01^**b**^	-5.3(-10.7–6.5)	-18.8(-25.1- -7.3)	<0.01^**b**^

Data are Median (Interquartile Range).

^**a**^ p<0.05 for differences between groups at baseline (Mann–Whitney U test).

^**b**^ p<0.05 for baseline to follow-up changes within group (Wilcoxon signed rank test).

SDR—selective dorsal rhizotomy

GDI—Gait Deviation Index

GC—Gait Cycle.

†Normalised to height.

‡ Rotation values: positive values internal; negative values external.

## Discussion

The aim of this study was to compare short-term changes in gait following SDR to a well matched control group who had no surgical intervention. The results showed that the short-term improvements following SDR are not due to natural development at the same age. On closer examination of the SDR group, those who had concomitant orthopaedic surgery demonstrated more improvements than those who had SDR only, though SDR only did result in improvements in some gait variables.

SDR (with or without orthopaedic surgery) resulted in more extended knees, reduced ankle equinus and reduced internal rotation of the tibia resulting in an improved foot progression angle during gait and improved normalised step length (Table 1, Fig 1). None of these changes were seen in the non-operated control group. There was a non-significant reduction in peak knee flexion during gait in the SDR group and clinical examination found a reduction in range in the rectus femoris between baseline and follow-up assessments. Improved peak knee flexion in swing has been considered one of the main outcomes following SDR [13, 14], and so it is unusual that peak knee flexion did not improve in the SDR group but it should be highlighted that these are short-term outcomes one year post surgery and so peak knee flexion may potentially be impacted on by residual weakness following surgery. The timing of peak knee flexion did improve following SDR Only consistent with the expected reduction in spasticity in the rectus femoris. However, this was not seen in the SDR and Orthopaedic Surgery group and it may be that orthopaedic intervention to the calf complex is resulting in residual reduction in ankle push-off power and impacting on knee flexion in swing.

Pre and post-operative clinical examination measures show that the SDR group demonstrated improved range in the hamstrings, gastrocnemius and rectus femoris post-operatively. This was not seen in the control group and potentially suggests that soft-tissue releases at or about the same time as the SDR contributed to some of the improvements in gait in the over-all SDR group. Examining those who had SDR only separately to those who had concomitant soft-tissue releases appears consistent with this and there were more positive changes in gait in those who had soft-tissue surgery at the same time (Table 2). This group demonstrated improved hip and ankle range of motion in the sagittal plane post-operatively which was not seen in the SDR only group. However, it must be noted that those groups were different at baseline analysis and those who had soft-tissue releases were more flexed at the hips and knees and so the decision to carry-out hamstring releases appeared to be appropriate. It is also notable that those who had soft-tissue surgery demonstrated increased anterior pelvic tilt post-operatively most likely due to hamstring release [15] and this was not seen following SDR only. Increased pelvic tilt has been demonstrated as an adverse effect of hamstring lengthening which can prevail at long-term follow-up and may be compensated for by increased lumbar lordosis which may be harmful over time [16].

The SDR-only group demonstrated improved normalised step-length, more extended knees at initial contact, improved knee range of movement, better timing of peak knee flexion and improved foot progression angles secondary to reduced internal rotation of the tibias. Comparison with appropriate control groups in this study suggests that SDR alone contributes to these gait improvement rather than concomitant soft-tissue lengthening or natural development. Improved step/stride lengths have previously been reported following SDR [17, 18]. That this improvement occurred following SDR only despite fewer improvements in gait kinematics compared to the SDR and orthopaedic surgery group potentially suggests that this is due to the generalised reduction in spasticity and stiffness. This may contribute to overall parent and child satisfaction as recent studies have noted that step length and knee extension at initial contact are bigger contributors to quality of life and oxygen consumption during gait than knee extension in mid-stance [19, 20].

As part of this study a matched control group were extracted from our database that had no surgical intervention. The changes in this group between baseline and follow-up analysis are interesting in that they highlight that gait kinematics are relatively stable at this age. There was a slight reduction in peak knee flexion and hip flexion/extension range but significantly, no increase in knee flexion in stance or tendency to crouch gait. This highlights the importance of considering natural progression in CP and has potential implications for treatment planning. It suggests that it is probably appropriate to delay Single Event Multi-Level Surgery (SEMLS) planning until a later age and in terms of SDR, the results potentially offer re-assurance that waiting until a child is older will not lead to any deterioration in gait.

There are a number of limitations to be considered in interpreting the results of this study. Firstly, we are reporting short-term outcomes at only one-year post operatively. As an implicit aim of SDR is to “optimize gait for the whole of life” we acknowledge that long-term outcomes are more important, particularly after the adolescent growth spurt [14]. However, establishing the short-term effects of SDR compared to an appropriately matched control group and also examining the effects of SDR only compared to SDR with orthopaedic surgery provides a more accurate baseline against which to compare longer term changes following SDR. We plan to continue to monitor this study cohort to assess these longer term outcomes. Secondly this study has primarily focussed on changes in gait kinematics and not referred to any impact on activity and participation. This reflects the fact that the SDR was not carried out in our centre so any assessment of quality of life or activity was not always available. We would advocate that a comprehensive assessment of SDR outcomes in CP should include outcomes in all ICF domains. Thirdly, we have not reported on clinical measures of muscle spasticity, as on retrospective review of our database this information was not always available. However, the effects of SDR on reducing spasticity have been well documented [1, 2] and so can be assumed. Fourthly, we took a strict approach to matching the groups at baseline for all relevant anthropometric and gait variables and while this did reduce the numbers in the study groups we feel that the results were more valid and the numbers are similar to other SDR studies. We did not specifically match the groups for gender but in all groups there were slightly more males than females (SDR group 59% male, No surgery Group 67% male; SDR Only group 63% male, SDR and Orthopaedic Surgery group 54% male) and so this is not felt to be a significant confounder.

Finally, as the SDR intervention (and associated orthopaedic procedures) was carried out in more than one centre and country it is possible that both selection criteria and surgical technique varied. Those assessed through the national pathway matched recognised selection criteria in ambulant CP i.e. ambulatory spastic diplegia, presence of significant spasticity interfering with mobility, good strength of lower limbs and trunk, no significant fixed contractures, good cognitive function and family support and no other movement disorders such as dystonia, ataxia or athetosis. [1, 9, 10]. However, a number of families’ self-funded travel to a centre in the United States for SDR and some of these patients at least may not have met these more widely accepted selection criteria. In terms of SDR surgical technique, the main difference was likely to be a single-level versus multi-level laminectomy approach. It has been shown that there are no significant differences in pain or time to mobilization between the techniques [21] and so it is unlikely that gait outcomes at one year post operatively would be different. Additionally, we did not always have full details on the orthopaedic surgical dose and so interventions were listed at the level of the muscle group rather than specific surgical procedures. We have demonstrated that those who had additional orthopaedic surgery were more flexed during gait before surgery and therefore had more improvements post-operatively. However, long-term results may be influenced by the type of orthopaedic surgery performed in the different surgical centres and one centre routinely carries out percutaneous hamstring lengthening’s potentially leading to increased injury to the muscle versus open lengthening [22].

In conclusion, we have demonstrated that SDR results in improvement in many gait parameters at short-term follow-up. While those who had concomitant orthopaedic surgery demonstrated more improvements in gait kinematics, comparison with a control group who had no surgery found that a number of improvements in gait were attributable to SDR only.
